# Gut bacteria and their metabolites may influence our decisions, intelligence, cognition, and consciousness

**DOI:** 10.1080/19490976.2026.2740906

**Published:** 2026-10-03

**Authors:** Leon M. T. Dicks, Alfred Botha, Carolina Pohl

**Affiliations:** a Department of Microbiology, Stellenbosch University, Stellenbosch, South Africa; b Department of Microbiology and Biochemistry, University of the Free State, Bloemfontein, South Africa

**Keywords:** Gut bacteria, metabolites, intelligence, cognition, consciousness

## Abstract

The human gut hosts approximately 38 trillion bacteria, representing 400 to 500 species. Gut bacteria are in constant communication with the central nervous system (CNS) via the enteric nervous system (ENS), the peripheral nervous system (PNS), the autonomic nervous system (ANS), the immune system, the enteroendocrine system (EES), and the vagus nerve (VN). Short-chain fatty acids (SCFAs), especially butyrate, strengthen the blood-brain barrier (BBB) and modulate inflammatory signaling, thereby affecting brain function and cognitive performance. Acetate enhances the synthesis of lipid-rich myelin that protects neurons. Signaling molecules that cross the gut wall may optimize synaptic signaling in the frontal cortex. Bacterial metabolites that stimulate inflammatory responses activate the hypothalamic-pituitary-adrenal (HPA) axis, thereby increasing glucocorticoid production. This may reduce activity in the prefrontal cortex and suppress higher-order thinking and decision-making. We discuss how signaling molecules produced by gut bacteria influence the limbic system (thalamus, amygdala, hypothalamus, and hippocampus), primarily involved in emotions, memory, and motivation. The HPA axis and the gut microbiota–oligodendrocyte axis (GMOA) are discussed in more detail. We ask whether interactions between signaling molecules produced by gut bacteria and the limbic system can affect human intelligence, cognition, consciousness, and decision-making, coordinated by the cerebrum (cerebral cortex), prefrontal cortex, and parietal lobe.

## Introduction

The human gut hosts approximately 38 trillion bacteria, representing 400 to 500 species.^1^ This is equivalent to the estimated three trillion (3.0 × 10^13^) cells in a person weighing 70 kg.^1^ According to Tierney et al.^2^ gut bacteria have approximately 46 million genes and, based on metagenomic studies, contribute over 99% of the genetic material in the human body.^1^ This gives bacteria a far-reaching genetic “networking” capacity that may influence our intellect, emotions, cognition, consciousness, and decision-making processes. It is thus not surprising that the role of gut microbiota on intestinal homeostasis, immunity, brain development, and mental health is well documented,^3^ and that the impact of diet, medication (especially antibiotics), age, lifestyle, and geography on the gut microbiome is covered in several reports.^4^ ^,^ ^5^ A number of studies investigated the influence of microbial metabolites on neurotransmitter dysfunction, inconsistent ATP production, and cognitive impairment.^6-8^ Evidence of a clear link between microbial dysbiosis and neurodevelopmental, neurodegenerative and psychiatric disorders such as autistic spectrum disorder (ASD), schizophrenia, Alzheimer’s disease (AD), major depressive disorder (MDD) and Parkinson’s disease (PD) prompted researchers to have a closer look at the gut-brain axis (GBA) and bidirectional communications between the CNS and the digestive system, neural, hormonal, immune, and metabolic pathways.^9^

Bonham et al.^10^ were the first to show that gut microbiota and their genes are associated with cognitive performance and brain structure. A total of 381 healthy children between 40 days and 10 years of age were enrolled in the study. Using a combination of multivariate linear models and machine learning (ML), the authors found large variations in microbial species in children older than 18 months. In this group of children, significant variations in cognitive function score (*R*2 = 0.0099, *q* < 0.01) and microbial gene functions (*R*2 = 0.0115, *q* < 0.001) were noted. Less variation in microbial taxa and genes, and less variation in cognitive functions, was observed in children younger than 6 months. Children older than 18 months and with higher cognitive function scores had higher cell numbers of *Alistipes obesi*, *Asaccharobacter celatus* (also known as *Adlercreutzia equolifaciens* subsp. *celatus*), *Gordonibacter pamelaeae,* and SCFA-producing probiotic species such as *Eubacterium eligens* and *Faecalibacterium prausnitzii.* ^10^ *A. equolifaciens* produces phytoestrogen-derived equol,^11^ a selective estrogen receptor beta (ERβ) agonist and a powerful antioxidant that modulates metabolic and hormonal processes^12^ and enhances the transcription of peroxisome proliferator-activated receptor gamma (PPARγ).^13^ Studies conducted on mice^14^ have shown that the activation of PPARγ resulted in a decline of Alzheimer's disease (AD), suggesting that it may prevent memory loss. PPARγ may thus support cognition by protecting nerve cells and reducing brain inflammation. *G. pamelaeae* produces the neuroprotective metabolite urolithin,^15^ ^,^ ^16^ which may, hypothetically, account for the higher cognitive scores in children older than 18 months.

Several metabolic pathways were either significantly enriched or depleted in children with higher cognitive ability scores.^10^ Genes encoding the degradation of acetate and propionate were significantly depleted in children younger than 6 months and with higher cognitive abilities. However, in children older than 18 months and with higher cognitive scores, an increase in acetate synthesis and propionate degradation was noted. These findings are noteworthy, as SCFAs play an important role in regulating the immune system and brain functions.^17^ Fewer genes encoding the synthesis of menaquinone (vitamin K) were observed in cognitively compromised children.^10^ Lower levels of menaquinone 4 (MK-4) were reported in children diagnosed with ASD. MK-4 is known for its neuroprotective abilities and may thus account for the increase in cognitive functions.^10^

An increase in genes encoding the synthesis of the amino acids glutamate and tryptophan was noted in children 18 months and older with higher cognitive scores.^10^ Glutamate regulates neuronal excitatory/inhibitory signaling.^18^ ^,^ ^19^ A decrease in Trp metabolism, normally associated with gut dysbiosis, and systemic inflammation, led to an increase in indoleamine 2,3-dioxygenase 1 (IDO1) production,^20^ associated with the production of neurotoxins, variations in serotonin production, and impaired intestinal barrier function,^21^ suggesting that the Trp-GBA may influence cognitive states and possibly also behavior and decision-making.

Bagheri et al.^22^ observed a notable correlation between plasma metabolites (signaling molecules) and the speed at which neurons process information. This may affect short-term and long-term cognitive functions, as shown in an experiment inoculating germ-free mice with human gut microbiota.^23^ Although the gut microbiome may contribute to cognitive functions and learning via the GBA,^9^ there is no direct evidence for this, and the concept of linking the gut microbiome to human intelligence, cognition, consciousness, and decision-making remains controversial. Human intelligence is a complex process that combines learning from experience, the ability to think abstractly, solve problems, and adapt to changing environments. Cognition is the collection of mental operations, perceptions, attention, memory, language, and reasoning, which are used to acquire, store, transform, and apply knowledge.^24^ Consciousness is a multifaceted and dynamic state, shifting between subjective states, internal experiences, feelings, or awareness of the self and the surrounding world.^25^ Decision-making is the cognitive process of evaluating options and selecting a specific course of action from alternatives to achieve a goal.^25^

Many studies linking the gut microbiome to human intelligence, cognition, consciousness, and decision-making are flawed because they are based on conflicting findings stemming from methodological mistakes, individual variability, and the challenge of proving direct causality versus correlation in cognitive and neurological studies. Observational studies often link specific microbial profiles to conditions such as autism, depression, or AD, but suffer from statistical and conceptual limitations that fail to establish direct causation^26^ ^,^ ^27^ Findings from murine studies often do not correlate with findings from clinical studies, mainly due to fundamental immune and metabolic differences,^28^ and the inconsistent use of diagnostic biomarkers and profiling methods.^29^ This emphasizes the need for human studies and more in-depth studies on microbial interactions, host metabolism, and neural networks, supported by multi-omics and neuroimaging.

In the present review, we discuss how signaling molecules produced by gut bacteria may influence our decisions, intelligence, cognition, and consciousness. We focus on the limbic system, specifically the thalamus, amygdala, hypothalamus, and hippocampus, which are primarily involved in emotions, memory, and motivation.^30^ The hippocampus, which plays an important role in the formation of short-term and long-term memory, learning, and spatial orientation, is particularly exposed to signaling molecules produced by gut bacteria.^31^ ^,^ ^32^ and informs the amygdala of physiological changes.^33^ The thalamus sorts sensory information, analyses changes in physiological conditions, and alerts the amygdala, which activates the sympathetic nervous system and the hypothalamic-pituitary-adrenal (HPA) axis. The latter governs the body’s hormonal stress response. We acknowledge that studies linking the gut microbiome to human cognition and decision-making have major limitations, including reliance on correlation rather than causation, small human sample sizes, and heavy dependence on animal models. We also understand that, due to the complexity of the human brain, concepts such as consciousness and intelligence are difficult to measure in the laboratory. Although human brain behavior cannot be attributed to a specific group of gut bacteria, it is important to examine how they communicate with the central nervous system via the complex network of neural, hormonal, immune, and metabolic pathways. Conclusions drawn from experiments conducted on laboratory animals are carefully evaluated.

## Major axes transferring signals of gut bacteria to the brain

### The gut-brain axis (GBA)

Gut bacteria are in constant bidirectional communication with the CNS via the enteric nervous system (ENS) and VN, collectively referred to as the GBA, reviewed by Dicks et al.^34^ The major signaling molecules produced by gut microbiota, and their communication with the CNS, are shown in Figure 1. Microbial signals may act through intestinal epithelial cells (IECs), enteroendocrine cells (EECs), immune cells, the VN, hepatic metabolism, and circulating mediators. Although all these routes are summarised in one figure, they act independently from each other. Signaling molecules that cross the GBB are transferred to the circulatory system via the hepatic portal vein (Figure 1). Signals reach the brain via the VN and enter the brain stem (Figure 1), from where they synapse onto elongated nucleus tractus solitarius (NTS) neurons A2, Prlh^+^, Glp1^+^, and Cck^+^.^35^ Although gamma-aminobutyric acid (GABA), serotonin (5-HT), dopamine (DA), and norepinephrine (NE) in Figure 1 are shown as neurotransmitters produced in the gut and crossing the intestinal barrier, it is important to note that they remain trapped in the peripheral circulation and cannot pass through the blood-brain barrier (BBB). Instead, they influence the brain indirectly (such as through the VN).

**Figure 1.**
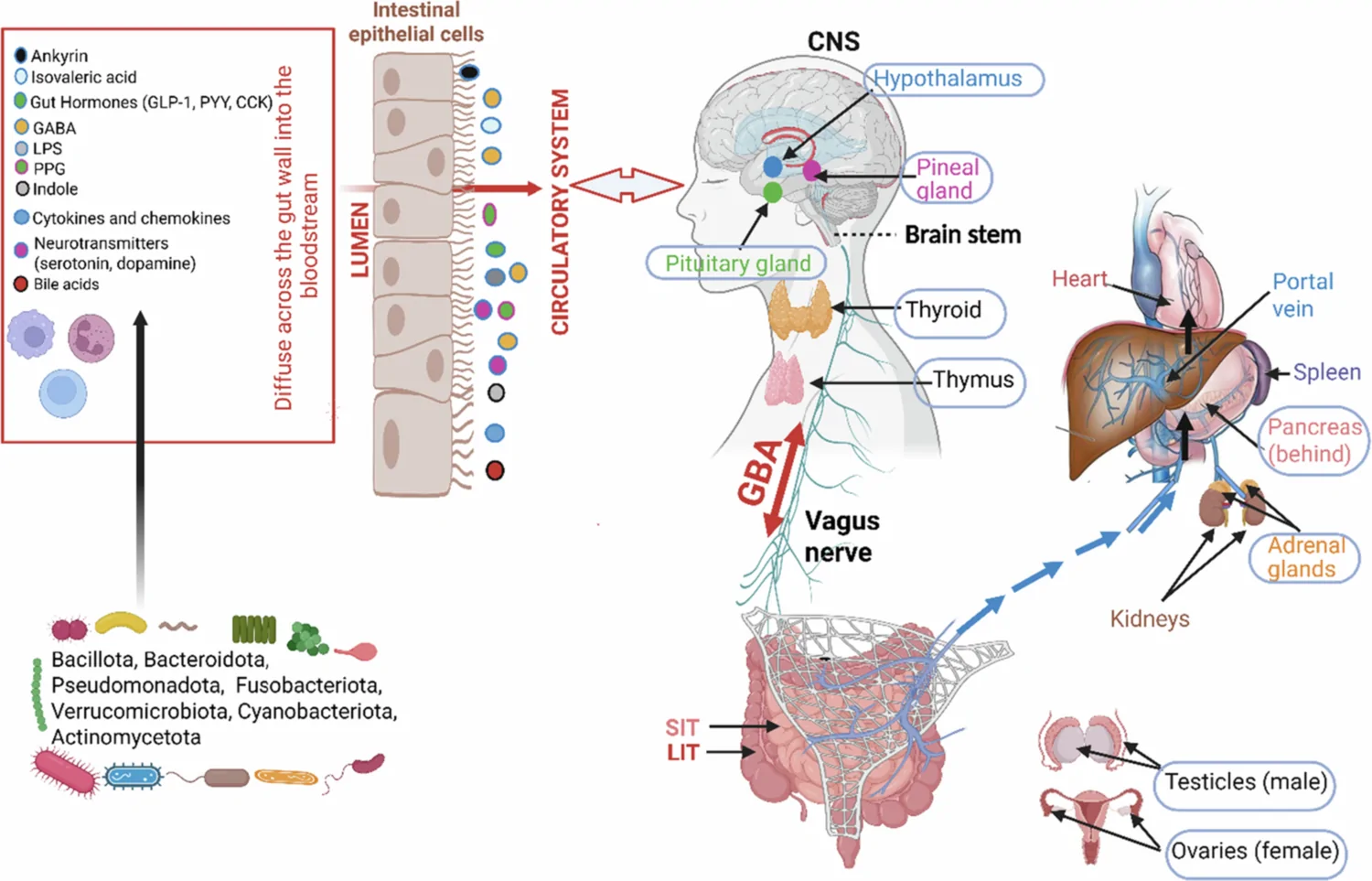
Examples of gut bacteria, key signaling molecules they produce (listed in the rectangle), and their communication with the central nervous system (CNS) via the enteric nervous system (ENS) and the Vagus nerve (VN), collectively referred to as the gut-brain axis (GBA). Organs involved in hormone production (the endocrine system) are encircled. Hormones produced by the hypothalamus travel through a primary capillary plexus to the portal veins, which carry them into the anterior pituitary. Hormones produced by the anterior pituitary (in response to releasing hormones) enter a secondary capillary plexus, and from there drain into the circulation. The anterior pituitary produces the growth hormone (GH), thyroid-stimulating hormone (TSH), adrenocorticotropic hormone (ACTH), follicle-stimulating hormone (FSH), luteinizing hormone (LH), beta endorphin, and prolactin. The pineal and thyroid glands produce melatonin and thyroid hormones, respectively. The thymus produces several humoral factors. The testicles produce testosterone, and the ovaries estrogen and progesterone. The pancreas produces insulin, and the kidneys renin and erythropoietin. The adrenal glands produce epinephrine and norepinephrine. GLP-1 = glucagon-like peptide 1, PYY = peptide tyrosine-tyrosine, CCK = cholecystokinin, GABA = gamma-aminobutyric acid, LPS = lipopolysaccharides (LPS), PPG = peptidoglycan, SIT = small intestinal tract, LIT = large intestinal tract. The figure was prepared using Biorender.com (assessed 15 May 2026).

Signaling molecules produced by gut bacteria also regulate immune responses and stimulate oligodendrocytes to produce a multilayered lipid membrane (myelin) sheath that protects neurons.^36^ The effects of signaling molecules on hormone production and neuronal myelination are covered in the following sections. All these systems need to be in perfect balance to control metabolism and maintain the gut microbiota in a balanced (homeostatic) state to regulate memory, learning, stress, and mood.

Experimental evidence supporting the impact of gut bacteria on neurogenesis and cognitive functions is discussed in several papers. Carlson et al.^37^ showed that there may be a link between gut bacteria and cognitive development. Certain gut bacteria, notably bifidobacteria, may improve our ability to recognize and discriminate between objects and modulate our cognitive processes, as shown with studies conducted on mice.^38^ Changes in the gut microbiome of mice influenced their social development, brain development, repetitive behavior, and cognitive functions.^39^ None of these physiological and neurological changes were observed in germ-free mice.^39^ The authors noted that similar impaired neurodevelopmental disorders were observed in patients suffering from schizophrenia and autism and hypothesized that these deficits may be associated with modulation of the immune system, specifically the release of cytokines, and changes in VN activity and neuroendocrine functions. Desbonet et al.^39^ have shown that treatment of mice with antibiotics following weaning increased anxiety, altered cognitive behaviors, and modulated neurological development in manners similar to those observed in germ-free mice. The authors concluded that dysregulation of the GBA in the post-weaning period may contribute to these disorders in adulthood, and attributed it to changes in neuromodulators such as noradrenaline, Trp, and the neuropeptides oxytocin and vasopressin. As mentioned elsewhere in this review, care should be taken not to make direct comparisons between animal studies and humans.

Studies conducted on human volunteers suffering from depression and anxiety^40^ have shown that changes in the gut microbiome cause systemic inflammation and alterations in SCFA production, leading to altered cognitive function. Foster et al.^41^ reported that cognitive functions in humans are dependent on the immune system and the production of neurotransmitters by gut bacteria. In another study, Dinan and Cryan^42^ showed that probiotics reduced inflammation and improved cognitive functions. Studies on rat PC12 cells showed that SCFAs can modulate genes encoding the cyclic adenosine monophosphate (cAMP) response element-binding (CREB) protein, which regulates the synthesis of catecholamine neurotransmitters, such as DA.^43^ ^,^ ^44^ Studies with rats showed that the conversion of DA to NE is initiated by SCFAs that activate the expression of tyrosine hydroxylase, the first reaction in the production of DA. This decreases the expression of DA-*β*-hydroxylase (DBH; EC 1.14.17.1), and thus the conversion of DA to NE.^45^ ^,^ ^46^ The hippocampus and striatum are especially susceptible to DA, which triggers a “neurotransmitter reward” response, and thus a change in mood.^47^ The rate at which DA is synthesized, including its conversion to NE, needs to be tightly regulated. A deficiency in DBH (thus elevated DA levels) may have a serious negative effect on the autonomic nervous system (ANS), which controls the regulation of blood pressure and body temperature.^48^

SCFAs regulate several other physiological functions in the human body, e.g., the maturation and functioning of microglia in the CNS,^49^ and the transmission of signals generated by 5-HT, GABA and DA to neurons.^50^ ^,^ ^51^ In EECs, SCFAs stimulate the release of gut hormones.^52^

Bile salts, combined with SCFAs, play an integral part in enterohepatic circulation, but are equally important in the regulation of neuronal pathways and signaling to the CNS.^53^ Studies with rats showed that free-fatty acid receptors FFAR2 and FFAR3 are expressed in the ENS, portal nerve and sensory ganglia,^54^ ^,^ ^55^ suggesting that SCFAs regulate the nervous system. This is supported by the induction of the nuclear phosphoprotein Fos in the dorsal vagal complex of the brainstem, the hypothalamus, and spinal cord.^54^ Fos expression is induced by serum, growth factors, tumor promoters, and cytokines. For more information on gut bacteria and neurotransmitters, the reader is referred to Dicks.^56^

Glutamate (Glu), released from presynaptic nerve terminals, is the principal excitatory neurotransmitter in the brain and plays a key role in memory storage.^57^ The adherence of Glu to ionotropic glutamate receptors (iGluRs), located on postsynaptic terminals, increases the transfer of Ca^2+^ through voltage-activated calcium channels (VACCs), and activates calcium calmodulin-dependent kinase (CaMK; EC 2.7.11.17). Glu also activates extracellular signal-regulated kinase (ERK, EC 2.7.11.24) and the CREB protein, all crucial for protein synthesis and maintenance of synaptic density. Excess Glu is taken up by glial cells via the EAAT1 and EAAT2 transporters. Glu is then converted to glutamine, which is transported back to presynaptic nerve terminals and converted to Glu by glutaminase (EC 3.5.1.2). The over-excitation of Glu (glutamate excitotoxicity) accelerates the progression of AD.^58^ For more information on glutamate excitotoxicity, the reader is referred to the review by Esposito et al.^59^

GABA is found in many brain regions and is released into the synaptic cleft upon depolarization of presynaptic neurons.^60^ GABA is also produced in glia (astrocytes) connected with pre- and post-synaptic neurons^61^ but different metabolic pathways.^62^ In GABA-expressing neurons, *α*-ketoglutarate, synthesized in the Krebs cycle, is converted to L-Glu by GABA *α*-oxoglutarate transaminase (GABA-T, EC 2.6.1.19) and decarboxylated to GABA by glutamate decarboxylase (GAD). In glial cells, Glu is not decarboxylated to GABA, as these cells do not express GAD. Instead, GABA is synthesized from *N*-acetylputrescine with monoamine oxidase B (MAOB, EC 1.4.3.4) as a key enzyme.^62^ GABA in glia is converted to succinic semialdehyde by GABA-T but also to glutamine, which is deaminated to glutamate before it re-enters the GABA shunt.^60^

In germ-free mice, significant neuronal development in the dorsal hippocampal region (essential for spatial learning and memory) was noted.^63^ In another study with infant germ-free mice,^64^ significant morphological changes of the amygdala and hippocampus were noted. The decline in neurogenesis could not be reversed by recolonizing the mice with gut bacteria,^65^ suggesting that neurogenesis is an early event and is controlled by the gut microbiome. In another study on mice,^66^ a decrease in hippocampal neurogenesis was prevented by pre-treatment with a combination of *Lactobacillus helveticus* and *Bifidobacterium longum*. Rolls et al.^67^ noted that mice deficient in TLR2 showed a reduction in neurogenesis and hippocampal volume, whilst an improvement in neurogenesis was observed in mice lacking TLR4 (genetic knockouts). However, mice lacking TLR4 showed a decrease in learning ability.^68^ The binding of lipopolysaccharides to TLR4 on neural progenitor cells (NPCs) inhibits proliferation and neuronal differentiation through MyD88 signaling.^67^ MyD88 is a myeloid differentiation primary response protein in humans that acts as a central communication hub for the innate immune system.^67^

Stimulation of the VN affects various cognitive functions, including learning and attention, verbal, working, and short-term memory, and decision-making.^69^ Electrical stimulation of the VN in mice led to improved memory, enhanced learning, and the recall of information.^70^ On a molecular level, VN stimulation is associated with changes in neurotransmitters such as GABA, Glu, 5-HT, DA, and NE.^69^ An increase in GABA led to an increase in synaptic connections between neurons in the brain and synaptic plasticity, both essential for learning and memory. Memory enhancement, accompanied by the stimulation of the VN, was noted when the levels of GABA and NE were increased.^69^ ^,^ ^71^ In mice, the vagal afferent nerves were activated when the diet was supplemented with GABA.^72^ Activation of the VN leads to an increase in the release of DA in areas such as the ventral tegmental area (VTA) and the substantia nigra, which are essential for neural plasticity related to reward and learning processes.^69^ ^,^ ^72-75^ A decrease in the population and diversity of gut bacteria (referred to as dysbiosis) alters VN stimulation and increases the number of pathogenic bacteria that produce signaling molecules that may lead to inflammation, oxidative stress, an imbalance in energy supply, increased cellular degeneration, and brain dysfunction.^76^ Both of the latter phenomena are instigated by an increase in the permeability of the intestinal mucosal barrier and the BBB.^77^

### The hypothalamic–pituitary–adrenal (HPA) axis

The hypothalamic-pituitary-adrenal (HPA) axis is the body's primary stress response system. The arcuate nucleus (ARC) of the hypothalamus and forebrain receives signals via the NTS (Figure 2, bottom) in the lower part of the brain stem, the medulla. Connection of the NTS with the neuromodulator nuclei is shown in Figure 2 (rectangle). The NTS connects with the nucleus basalis of Meynert in the basal forebrain, raphe nuclei in the midbrain, pons, and medulla; and the locus coeruleus in the pons. These nuclei regulate vital homeostatic functions such as feeding behavior, appetite,^78^ the release of hormones (e.g., cortisol, oxytocin, and vasopressin), and the production of glutamate and GABA, acetylcholine, NE, and 5-HT. They also distribute the hormones to the cortex, subcortical nuclei, and spine.^35^ Although Gruber et al.^35^ did not find evidence of a link between HPA dysfunction and cognition, they concluded that a more responsive HPA axis may be associated with an increase in cognitive abilities, especially at old age. Sharan and Vellapandian,^79^ however, found that chronic HPA axis dysregulation and an overproduction of cortisol can impair memory, decision-making, and long-term cognitive functions.

**Figure 2.**
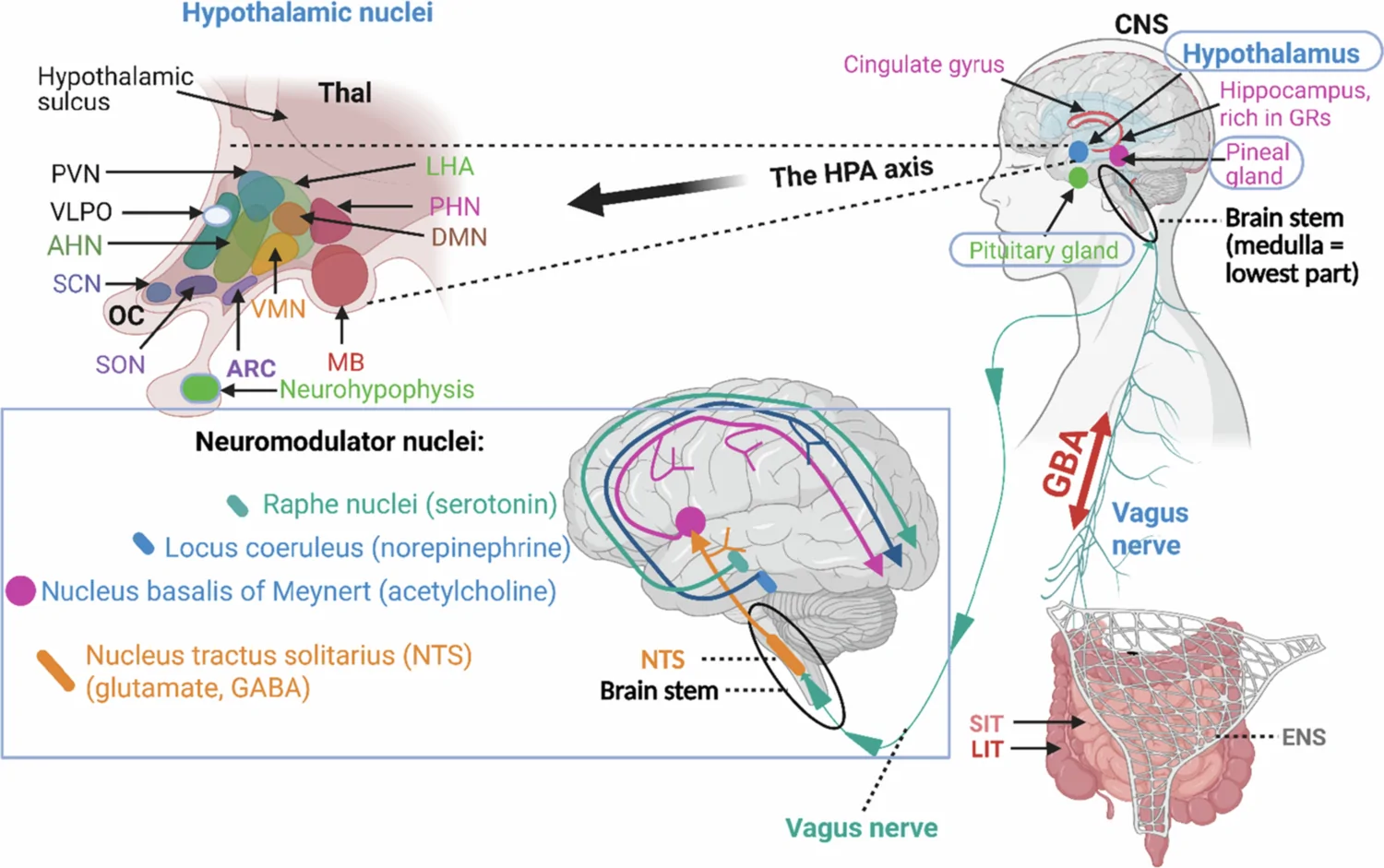
Right side: Intestinal microbiota (including bacteria) in the large intestinal tract (LIT), not so much the small intestinal tract (SIT), communicate with the central nervous system (CNS) via the Vagus nerve (VN), and connect to the nucleus tractus solitarius (NTS) in the brain stem. Bottom of diagram: On entering the medulla (the lowest part of the brain stem), the VN synapses bilaterally in the nuclei of the NTS. The NTS connects to the key neuromodulator nuclei (listed in the rectangle): The nucleus basalis of Meynert in the basal forebrain, raphe nuclei in the midbrain, pons, and medulla; the locus coeruleus in the pons; and the raphe nuclei positioned symmetrically along the midline of the brainstem. The NTS stimulates the production of glutamate and gamma-aminobutyric acid (GABA); the nucleus basalis acetylcholine; the locus coeruleus norepinephrine; and the raphe nuclei serotonin (listed in the rectangle). These hormones are distributed to the cortex, subcortical nuclei, and spinal targets. Top left of diagram: The hypothalamus senses and integrates signals related to the body’s nutritional and metabolic status and regulates feeding behavior and nutrient metabolism. In response to stressors, excitatory neuronal inputs activate the hypothalamic-pituitary-adrenal (HPA) axis and stimulate the production of hormones. Activation via excitatory neuronal inputs to the paraventricular nucleus (PVN) of the hypothalamus releases corticotropin-releasing hormone (CRH) and arginine vasopressin (AVP). CRH induces corticotropes in the anterior pituitary to stimulate production of adrenocorticotropic hormone (ACTH). ACTH is released into the blood and is transported to the adrenal glands. In the adrenal glands, ACTH initiates the synthesis of cortisol (CORT), which is then released into the circulatory system and reaches multiple tissues, e.g., the lungs, heart, and muscles. A decrease in CORT is associated with increased inflammation, which could contribute to mental health conditions, behavioral changes, or emotional complications. This usually arises as a direct or indirect result of a primary illness, traumatic brain injury, severe infection, or trauma [e.g., prolonged psychiatric conditions such as post-traumatic stress disorder (PTSD), panic disorder, phobias, and major depressive disorder]. Signaling molecules and energy-related (leptin, insulin, ghrelin) peptides access target neurons in the arcuate nucleus (ARC) through a regulated passage that involves highly specialized hypothalamic glial cells (tanycytes, not shown). DMN = dorsomedial nucleus, ENS = enteric nervous system, GRs = glucocorticoid (cortisol) receptors, LHA = lateral hypothalamic area, MB = mammillary body, OC = optic chiasm, PHN = posterior hypothalamic nucleus, SCN = suprachiasmatic nucleus, SON = supraoptic nucleus, Thal = thalamus, VLPO = ventrolateral preoptic nucleus, VMN = ventromedial nucleus. The figure was prepared using Biorender.com (assessed 15 May 2026).

The hippocampus (a seahorse-shaped structure located deep within the brain’s temporal lobe, Figure 2, right side) is highly sensitive to changes in the HPA axis, due to a high density of glucocorticoid (cortisol) receptors (GRs) in this region. The expression of GRs is, however, reduced during dysbiosis, increasing the abundance of pathogenic bacteria.^80^ Patients suffering from acute Crohn’s disease (chronic inflammation) may have a dysfunctional HPA, impairing the body's ability to regulate inflammation and stress responses.^81^ An overactive HPA (increased cortisol production) becomes resistant to glucocorticoids and loses the ability to inhibit inflammation. The release of corticotropin-releasing hormone (CRH) increases intestinal permeability and may lead to leaky gut syndrome, thereby amplifying inflammatory responses.^82^ An increase in interleukin-17A (IL-17A) from T helper 17 (Th17) cells in the lamina propria leads to an increase in the release of aged neutrophils into the bloodstream. Mice subjected to chronic stress showed a decrease in beneficial bacteria such as *Lactobacillus*, and an increase in kynurenine. Treatment with *Lactobacillus reuteri* controlled kynurenine levels, normalised (balanced) the gut microbiome, and ameliorated behavioral abnormalities.^83^ Repressing the production of glucocorticoid and blocking of IL-17A alleviated stress and improved learning.^84^

Germ-free mice with an impaired HPA displayed changes in the prefrontal cortex, hippocampus, and amygdala.^85^ Long-term exposure to high levels of glucocorticoids leads to decreased activity in the prefrontal cortex, and thus to lower-order thinking and decision-making. At the same time, activity in the amygdala (which regulates emotional responses and survival instincts) increases.^86^ For further information on the HPA axis, the reader is referred to the review by Alavian and Safaeian.^87^

### The gut microbiota–oligodendrocyte axis (GMOA)

The gut microbiota–oligodendrocyte axis (GMOA, Figure 3) refers to the association between gut microbiota and the multilayered lipid membrane myelin produced by oligodendrocytes in the CNS. The sheathing of nerve axons allows for the leaping and sudden propagation of action potentials^88^ and increases nerve impulse velocity as much as 100-fold.^89^ Myelin is produced by oligodendrocytes in the CNS and Schwann cells in the PNS.^90^

**Figure 3.**
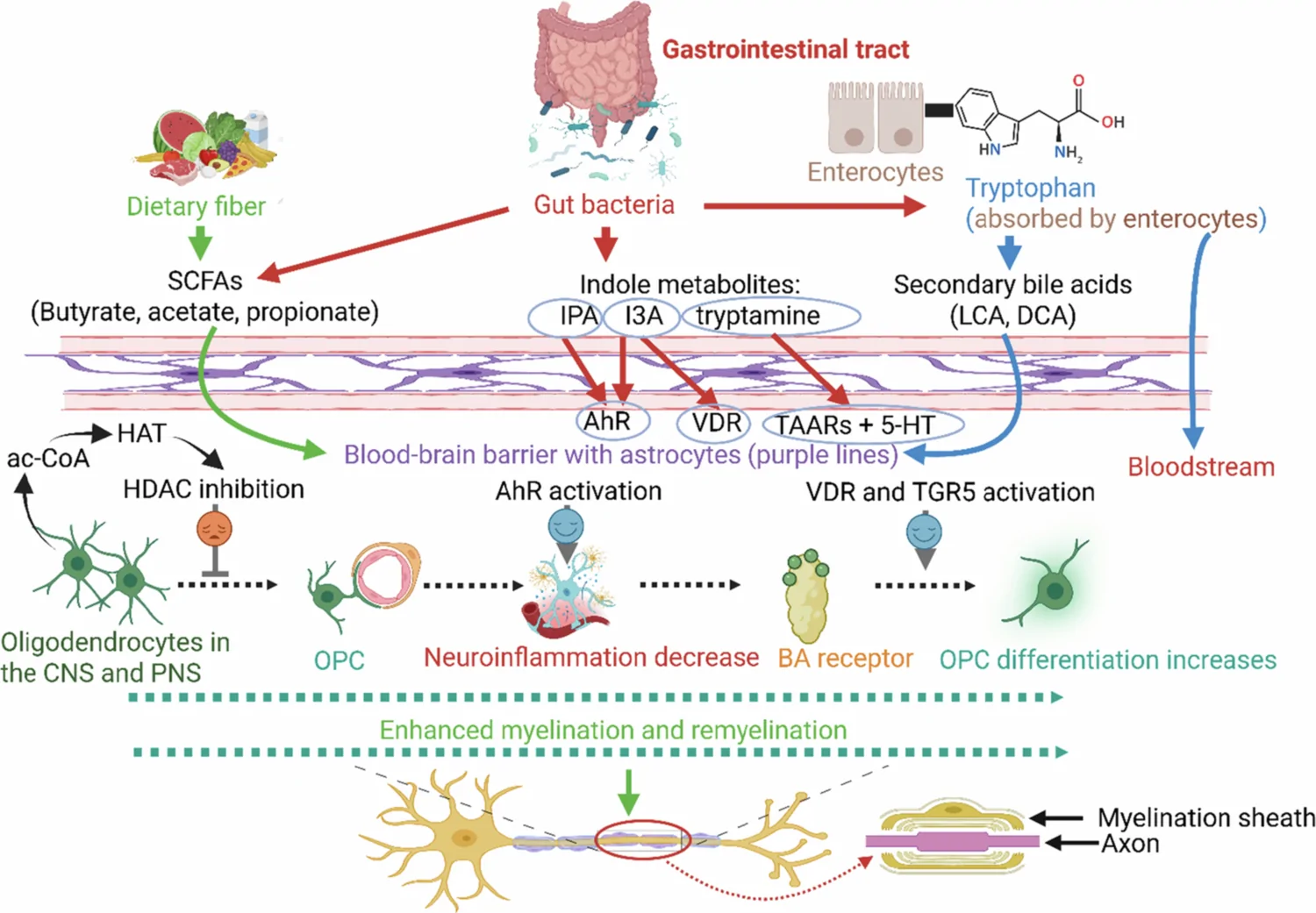
A summary of the gut microbiota–oligodendrocyte axis (GMOA), also known as the gut-myelin axis. Short-chain fatty acids (SCFAs), especially acetate, produced by gut bacteria from the metabolism of dietary fiber, cross the blood-brain barrier (BBB). Acetyl-CoA synthetase in oligodendrocytes (located in the central nervous system, CNS), and Schwann cells in the peripheral nervous system (PNS), converts acetate into acetyl-CoA (ac-CoA), which is used in lipid biosynthesis pathways to form and maintain the myelin sheath that surrounds neurons. Ac-CoA provides acetyl groups for histone acetyltransferases (HATs) and inhibits histone deacetylase (HDAC). Acetylation of histone proteins relaxes the chromatin structure and activates the transcription of genes encoding myelin production. Indole-3-propionic acid (IPA) and indole-3-aldehyde (I3A), produced by gut bacteria, cross the BBB. IPA, a potent antioxidant and a strong ligand for the aryl hydrocarbon receptor (AhR), reduces neuroinflammation, lowers amyloid-beta aggregation, and increases neuroprotective metabolites such as kynurenic acid. I3A acts as an agonist for the AhR and activates the AhR pathway. I3A thus regulates immune responses and neuroinflammation, which helps to suppress systemic and central nervous system autoimmunity. I3A binds to the vitamin D receptor (VDR) to regulate mucosal immunity, intestinal homeostasis, and epithelial barrier integrity. Tryptophan, an essential amino acid, is absorbed by enterocytes in the small intestine, from where it enters the bloodstream. Colonic bacteria convert tryptophan into indole and its derivatives (IPA and tryptamine). Tryptophan prevents the destruction of myelin, thus preventing neuroinflammation. Tryptamine binds to trace amine-associated receptors (TAARs) and serotonin (5-HT) receptors, and functions as a neuromodulator of the CNS. LCA = lithocholic acid, DCA = deoxycholic acid, TGR5 = Takeda G protein-coupled receptor 5 (also known as GPBAR1 or G protein-coupled bile acid receptor 1), OPC = oligodendrocyte precursor cells, BA = bile acid. The figure was prepared using Biorender.com (assessed 15 May 2026).

SCFAs, especially acetate, produced by gut bacteria from the metabolism of dietary fiber, cross the BBB. Ac-CoA synthetase in oligodendrocytes (located in the CNS), and Schwann cells in the PNS, converts acetate into ac-CoA, which feeds directly into the lipid biosynthesis pathways required to form and maintain the myelin sheath.^88^ ^,^ ^90^ The acetyl groups from ac-CoA serve as substrates for histone acetyltransferases (HATs). Acetylation of histone proteins relaxes the chromatin structure and activates the transcription of genes encoding myelin production. The myelin (myelin sheath) provides structural support to axons.^90^

An increase in acetate, produced by gut bacteria, enhanced the synthesis of myelin lipids in a rodent model.^91^ Dysbiosis related to the depletion of acetate-producing bacteria may thus lead to reduced myelin production and dysfunctional neurons.^88^ Changes in the myelination of neurons in areas of the brain involved with memory and learning will thus affect cognitive functions, social behavior, memory, and decision-making. Humans experience late myelination of frontal cortex axons in their third decade of life, making them vulnerable to external factors such as microbial metabolites.^92^ Recent research on the GBA indicated that demyelination may be reversed by increasing the population of certain gut bacteria. Further research on the GMOA may shed more light on multiple sclerosis (MS), which affects approximately 2.8 million people worldwide.^93^

IPA and I3A, produced by gut bacteria, cross the BBB. IPA is a potent antioxidant and a strong ligand for the aryl hydrocarbon receptor (AhR) and reduces neuroinflammation, lowers amyloid-beta aggregation, and increases neuroprotective metabolites such as kynurenic acid.^94^ I3A, synthesized by gut bacteria, acts as an agonist for the AhR. By activating the AhR pathway, I3A regulates immune responses and neuroinflammation and plays a critical role in suppressing systemic and central nervous system autoimmunity.^95^ The interaction between I3A and the vitamin D receptor (VDR) regulates mucosal immunity, intestinal homeostasis, and epithelial barrier integrity.^96^

Tryptophan is an essential amino acid (not synthesized by the human body) and is supplied from dietary sources such as meats, eggs, and seeds.^97^ Specialized intestinal epithelial cells (enterocytes) in the small intestine absorb tryptophan and transport it to the bloodstream.^97^ Tryptophan that is not absorbed reaches the colon, where it is co-metabolised by gut bacteria and the host. Gut bacteria convert tryptophan into indole, IPA, and tryptamine. Between 1 and 2% of the tryptophan is converted into serotonin by enterochromaffin cells. Most of the tryptophan is metabolized via the kynurenine pathway, from where it regulates the immune system and prevents the destruction of myelin (thus preventing neuroinflammation).^98^ Tryptamine binds to trace amine-associated receptors (TAARs) and serotonin (5-HT) receptors to regulate dopaminergic, serotonergic, and glutamatergic systems. Tryptamine, thus, functions as a neuromodulator of the CNS. For further information on MS and GMOA, the reader is referred to the review by Torres et al.^88^ and Tang et al.^99^

Hoban et al.^100^ discovered that certain gut bacteria stimulate the myelination of nerves in the prefrontal cortex (PFC) and stated that “appropriate cortical myelination relies on the presence of a functional microbiota during critical windows of neurodevelopment”. We know gut bacteria stimulate synaptic growth and plasticity, as clearly evident in patients with neurological disorders such as PD and AD.^101^

## How does bacterial “intelligence” influence our intelligence?

Bacterial “intelligence” may be defined as the ability to adapt to external factors and practise competitive coherence. The latter refers to connectivity-based competition between a current (developing) phenotypic and genotypic state and subsequent states. For example, a cell that expresses a set of genes encoding, e.g., heat shock and cold shock proteins,^102^ is likely to be outcompeted by a next generation of cells that requires only one of the two gene sets. Thus, for bacteria to maintain a competitive coherent state, the cell must adapt to internal and external conditions and overcome selective pressure. If cells make the wrong decision (imposed upon by, e.g., a sudden change in molecular stimuli), they will not evolve and survive. Bacterial sentience is defined as “feelings” or behavior based on “experiences,” and cognition as the ability to receive, process (learn), store, and act (decide) based on observations (e.g., of the environment) and memory.^103^ Biofilm formation is a good example displaying intelligence, sentience, and cognition. In a biofilm, genetically related cells exposed to the same environment develop new phenotypic characteristics (known as bet-hedging), alter their phenotypic characteristics to explore multiple environmental niches (referred to as stochastic switching), and display individualistic nutrient uptake.^104^ Collective decision-making is a coordinated, social process, often driven by changes in chemical signals that trigger quorum sensing (QS) at a specific cell density. Gram-negative bacteria may produce several forms of AHLs,^105^ each detected by specific LuxR (luciferase regulator) receptors^106^ ^,^ ^107^ AHLs pass through the cell membrane and bind directly to soluble receptor proteins. The SdiA (suppressor of division inhibition) QS receptor, found in *Escherichia coli* and *Salmonella* spp., activates transcription of the *ftsQAZ* gene cluster, thereby increasing production of the cell-division protein FtsZ. LuxS (S-ribosylhomocysteine lyase) cleaves the thioether bond of S-ribosylhomocysteine (SRH) to produce 4,5-dihydroxy-2,3-pentanedione (DPD), which, due to its instability, immediately cyclizes and rearranges into various AI-2 derivatives.^108^ AI-3, combined with Epi and NE, forms the AI-3/Epi/NE pathway, which coordinates gene expression, motility, and virulence.^109^ Enterohemorrhagic *E. coli* (EHEC) uses the AI-3/Epi/NE pathway to upregulate the locus of enterocyte effacement (LEE) pathogenicity island, promoting the formation of lesions in IECs^110^ For further information, the reader is referred to the review by Dicks.^111^ and Dicks et al.^108^

Bacteria developed a complex “toolkit” by assessing the significance of the signals they receive, evaluating each signal, and responding in their best interest.^112^ The spectrum of molecular signals bacteria respond to, and the machinery used to process signals and respond to each, differ for each species and strain. Key signaling mechanisms of bacteria are quorum sensing (QS), two-component systems (TCSs), and mechanosensing (e.g., the triggering of cyclic diguanylate, c-di-GMP).^113^ Memory-based decisions are not made on changes in individual molecules but on persistent changes in bacterial cells, e.g., DNA methylation, histone modifications, and changes in glycolysis.^114^ ^,^ ^115^

The survival of bacteria (living cells in general) depends on the ability to synchronize gene transcription and translation (mRNA, tRNA), express polyamines, functional proteins and enzymes, produce macromolecules such as fatty acids, adenosine triphosphate (ATP), cytidine triposphate (CTP), cyclic adenosine monophosphate (cAMP), and regulate the flow of micro molecules, such as calcium, iron, zinc, etc. Cells “learn” from DNA regions transcribed upstream, gene activators and repressors, mRNA terminators, and post-translational modifications to avoid next-generation mistakes. The adaptation and reaction of bacteria to the gastrointestinal environment is far more complex than an “action-reaction” process. This is especially evident with cells grouped in a colony or biofilm. Grouped cells self-/re-organize according to their nutritional requirements and form “hyper structures” such as cytoskeletal filaments; elongation factor Tu (EF-Tu); CTP synthase; RNases; proteinaceous organelles (large, polyhedral, protein-based assemblies) that sequester metabolic intermediates; chemoreceptors; lipid rafts; acidocalcisomes; polyphosphate granules; E1 proteins that unwind DNA ahead of the replication fork; enzyme complexes, e.g., involved in phosphorylation; membrane proteins; and proteins involved in DNA replication, transcription, transfer of signaling molecules across cell membranes, and bacteriophage adhesion.^116^

The “intelligence” of gut bacteria is perhaps best understood by analysing the genetic abilities of a simple (but intelligent) bacterium such as *Escherichia coli*. The cell’s growth, metabolism, colonisation of the gut wall (intestinal epithelial cells, IECs, and mucus layers), replication, and survival are controlled by more than 300 transcriptional regulators.^117^ This allows *E. coli* to degrade different sources of carbon, nitrogen, phosphorus, and sulphur, withstand changes in oxygen tension, temperature, osmotic pressure, and pH, and develop resistance to, e.g., heavy metals, free radicals, and antibiotics.^117^

Bacteria transfer molecular signals using one-, two-, and multi-component systems located in the cell membrane. TCSs are often referred to as a “nano-brain”, thereby implying that they represent neuronal networks of higher life forms. Neuronal connections are limited to synapses on dendrites. In bacteria, these connections are represented by nanotubes and nanowires.^116^ With such a complex signal-transducing network, metabolically active bacteria are constantly in a state of competitive coherence and relate current phenotypic and genetic states to previously encountered scenarios.^116^ Taking all these functions into consideration and keeping in mind that they are on average 1-2 μm in length, one may argue that the genetic capabilities of bacteria are superior to those of humans.

There is a close relationship between gut bacteria and brain development, particularly concerning learning, attention, and memory.^118^ However, research linking human intelligence, cognition, consciousness, and decision-making to the gut microbiome is complex (Figure 4) and often borders on assumptions rather than experimental facts pointing to specific agents or signalling molecules. Tillisch et al.^119^ claimed that probiotic bacteria alter human cognitive behavior. Although a valuable observation, the reasons and mechanisms involved have not been clarified. Studies on patients with irritable bowel syndrome (IBS), which we know is caused by many factors, including an imbalance in gut microbiota, revealed evidence of a stress-related impairment in visuospatial memory,^120^ defined as a cognitive system responsible for temporarily storing, maintaining, and manipulating visual and spatial information, such as the shapes of objects, their colors, and their locations in an environment.^121^ Research approaches to study the influence of gut microbiota on human intelligence, cognition, consciousness, and decision-making are listed in Table 1.

**Figure 4.**
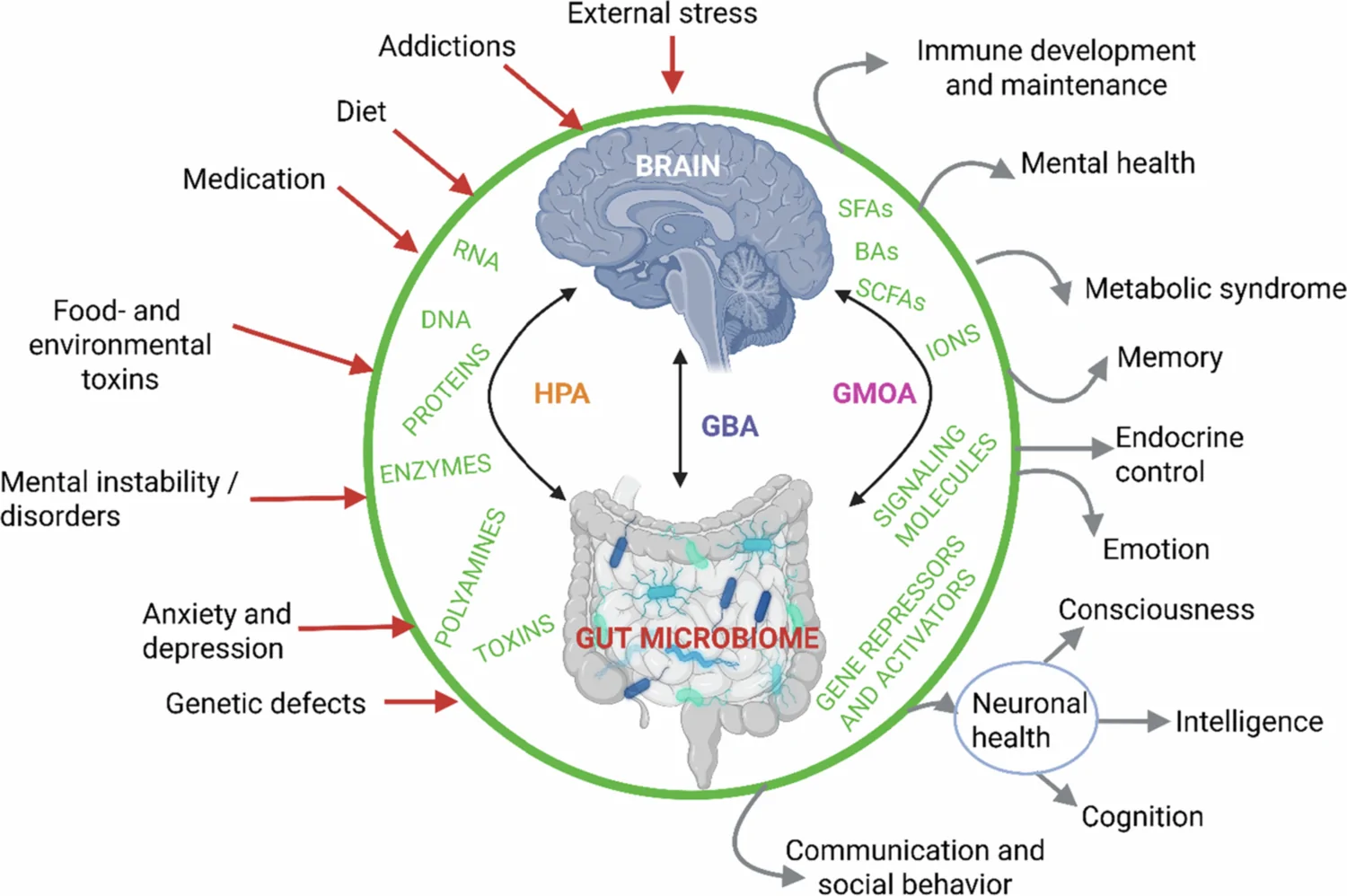
Gut bacteria influence our intelligence, cognition, and consciousness in numerous ways. Signaling molecules produced by gut bacteria and the brain (listed in green) communicate via three main axes: the GBA (gut-brain axis), the HPA (hypothalamic–pituitary–adrenal axis), and the GMOA (gut microbiota–oligodendrocyte axis). On the left of the circle are examples of external factors that may affect brain health. On the right of the circle are examples of symptoms and conditions that may develop as a result of communications via the GBA, HPA, and GMOA. The figure was prepared using Biorender.com (assessed 15 May 2026).

**Table 1.** Research approaches to study the influence of gut microbiota on human intelligence, cognition, consciousness, and decision-making.

Research approach	Intelligence and cognition	Consciousness and awareness	Decision-making	Key strengths and limitations	References
**Interventional**	Diet variations and probiotic preparations yield mild-to-moderate improvements in verbal memory, visuospatial processing, and executive flexibility under stressful conditions	Data based on proxy states such as vigilance and alertness. Studies on major shifts in core consciousness are limited	Changes in diet, prebiotic and probiotic supplements alter risk tolerance, reward processing, and social fairness perception	Strengths: Establishes human causality and is clinically relevant. Limitations: Short timeframes, subtle effects, and high individual variability	[122–125]
**Observational**	An increase in alpha-diversity correlates with better executive functions and reduces the risk of mild cognitive impairment	Cross-sectional studies link structural networks to baseline brain connectivity and brain waves, assisting in the study of physical pathways and the development of mental awareness	Variations in gut microbiome composition and taxonomic abundance profiles link to decision-making and behavioral impulsivity. Bacteria produce neurotransmitters and affect metabolic pathways that influence self-control, risk-taking, and social cooperation	Strengths: Identifies real-world patterns and long-term associations. Limitations: Cannot prove causation, and is heavily confounded by lifestyle and diet	[126–131]
**Germ-free/antibiotic models**	Germ-free rodents show reduced brain cell growth in the hippocampus, weaker brain connections, and poor memory, which may be remedied with probiotic supplements	Lack of conventional sensory-autonomic drive causes atypical baseline alertness and stress reactions, leading to unusual brain activation patterns, changed physiological responses, and altered emotional processing	Absence of microbiota disrupts anxiety-like behavior and risk assessment in maze tests. Gut bacteria help shape how the brain reacts to stress	Strengths: Proves absolute necessity of microbes for normal brain development. Demonstrates the impact of sudden microbial loss on the brain. Limitations: Anatomical differences, cannot fully replicate human consciousness, confounding off-target toxicity, transient effects	[87,132–135]
**Fecal microbiota transplant (FMT) studies**	FMT from donors with cognitive impairment or metabolic conditions transfer behavioral deficits, altered brain metabolism, and neuro-inflammatory traits to recipients via the GBA	Transfer of donor gut bacteria modifies the CNS and behavior via the GBA. Rodent studies demonstrate shifts in brain electrical activity, neurogenesis, and synaptic plasticity. Human studies are primarily focused on conditions such as hepatic encephalopathy and metabolic syndrome (MS)	Specific behavioral traits, including anxiety, depression, impulsivity, and compulsive or reward-seeking habits, can be transferred via FMTs from the donor to the recipient	Strength: Proves that specific behavioural phenotypes are transmissible. Limitations: High variability in donor material, and safety risks in human trials	[136–139]
**Mechanistic metabolite/receptor Studies**	Short-chain fatty acids (acetate, propionate, butyrate) and tryptophan metabolites cross the blood-brain barrier, modulating the growth of microglia, increasing brain-derived neurotrophic factor (BDNF), and oxidative phosphorylation in neurons	Vagal nerve signaling and systemic cytokine modulation regulate central arousal thresholds necessary for alert states.	Microbial transformation of tyrosine and phenylalanine alters central DA and serotonin precursors, which may influence the choice between immediate smaller rewards and delayed larger rewards	Strength: Pinpoints the exact chemical signals that alter neural circuits. Limitation: Oversimplifies complex microbial ecosystems to single molecules	[140–143]
**Conceptual and philosophical interpretations**	Microbial genomes may be viewed as a distributed cognitive extension. Human agency and rational optimization cannot be cleanly separated from the symbiotic enteric ecosystem	Large-brain evolution and high intelligence as energetically enabled by specialized microbial metabolic pathways that optimize glucose-to-ATP conversion in the frontal cortex	Higher-order consciousness and free decision-making emerge purely from intrinsic central nervous network topology or are permanently coupled with visceral homeostatic feedback loops	Strengths: Connects disparate fields, and drives future hypothesis generation. Limitations: Lacks empirical testing and is speculative by nature	[144–148]

Gut bacteria regulate hippocampal neurogenesis and may thereby affect learning and memory.^65^ Disturbances in the microbiota led to abnormal neurogenesis and cognitive functions, as shown with a significant increase in dorsal hippocampal neurons^63^ and morphological changes in the amygdala and hippocampus of infant mice. The recolonization of mice after infancy did not stimulate neurogenesis, suggesting that the regulation of neurogenesis by the gut microbiota must occur early in life.^65^ A decrease in neurogenesis, due to stress, could be prevented by pretreatment with probiotic strains of *Lactobacillus helveticus* and *Bifidobacterium longum.* ^66^ A deficiency in TLR2 reduced neurogenesis and hippocampal volume, whereas TLR4 knockout mice showed an increase in neurogenesis,^67^ accompanied by a decrease in learning ability.^68^ The reason for these changes was attributed to the disruption of a MyD88-dependent Toll-like receptor 4 (TLR4) signaling cascade when MyD88 was inhibited. The blocking of MyD88 disrupts the binding of LPS to TLR4 on neural progenitor cells (NPCs) and deactivates NF-κB.^67^ This prevents the downstream transcriptional programs that normally protect cells or drive inflammation, resulting in reduced inflammatory signaling and altered neurodevelopment. These studies demonstrate the role of bacteria and their metabolites on neurodevelopment and their potential impact on the regulation of cognitive function. It is, however, interesting to note that when the developing of neurons in the hippocampus is suppressed during early life, communication between the existing neurons increase to retain or improve memory.^149-151^

Most studies failed to identify specific bacterial molecules or chemical reactions that impact learning and memory. The first breakthrough came with the report from Kunze et al.^152^ showing that *Lactobacillus reuteri* repressed the opening of calcium-dependent potassium channels and, by doing so, decreased the hyperpolarization in sensory colonic AH (after hyperpolarization) neurons in the ENS. AH neurons experience a prolonged drop in membrane potential for up to 20 secs, due to changes in calcium-dependent potassium channels.^153^ They act as intrinsic primary afferent neurons (IPANs), focused on detecting physical and chemical changes in the intestinal tract, e.g., the expansion or contraction of the gut wall, nutrient levels, and changes in pH.^153^ In humans, serotonin- and GABA-producing gut microbiota regulate the maturation of the ENS and relays vital sensory signals back to the CNS via the ENS, PNS, and VN.^154^ O'Mahony et al.^155^ claimed that altering the gut microbiota of rats early in life alleviated visceral pain in fully grown rats, but did not affect cognitive or anxiety-related behaviors. In a subsequent study,^156^ it was shown that the response to pain was due to an increase in activity-related transcriptional pathways in the amygdala. Dendrites in the amygdala and hippocampus of mice with an imbalanced gut microbiome were notably different from those in the control group,^64^ suggesting that the functioning of neurons in these areas of the brain depends on a balanced gut microbiome.

Neurotransmitters such as GABA, DA and tryptophan derivatives (e.g., serotonin) enter the bloodstream directly, bypassing neurons of the ENS/PNS and communicate directly with distant organs.^157^ These metabolites reach the liver through the portal vein and interact directly with receptors on cells in the pancreas, kidneys, lungs, and heart.^158^ Cytokines, released from immune cells that have been stimulated by bacterial signaling molecules, activate microglia of the CNS.^159^ This “bacteria-mind continuity” supports the idea of an endless cooperation between biological and mind processes, suggesting that consciousness may be deeply rooted in the physiology of bacteria and humans, supporting the theory of a “holobiont mind”^160^ (Figure 5), which re-defines human intelligence, cognition, and consciousness. Mark Rowlands^161^ referred to this hypothetical extracranial process as “4E cognition”, with reference to the four “Es” in cognition, i.e., “embodied”, “embedded”, “enacted”, and “extended”). Embodied cognition defines our ability to think, learn, and remember based on how we interact with the physical world. Embedded cognition refers to the use of tools and social structures to support cognitive functions. Enacted cognition is defined as information gained via dynamic interactions (e.g., the human body and its environment). The GBA, HPA, and GMOA fall within this definition. Enacted cognition is often referred to as enactivism, which relates to embodied cognition, combined with a sense-making process that involving brain, body, environment, and subjective experience. Extended cognition is more of a philosophical concept and is described as functions of the mind and cognitive processes spilling over to the environment. This suggests that physical objects, sensory experiences, environments, and people influence our thinking, learning, and memory. This implies that the brain is a computational organ influenced by many factors. Not addressed in this hypothesis is the influence of the gut microbiome and the signaling molecules they produce on thinking, learning, and memory. This suggests that gut bacteria may have a more profound influence over our intelligence, cognition, consciousness, and decision-making than originally anticipated.

## Conclusions

Human intelligence, cognition, consciousness, and decision-making are not solely “brain-bound”. The signaling molecules and neurotransmitters produced by gut bacteria, e.g., 5-HT, DA, and GABA may affect our responses to internal and external stimuli. Gut bacteria may play a crucial role in neurological development, regulating brain chemistry, mood, social behavior, and stress responses. Bacterial interactions with the CNS are driven by the GBA, HPA, and GMOA. Gut bacteria act as an “organ” in a complex, symbiotic ecosystem, participating in the regulation of metabolism, biochemical pathways, immune functions, and neurological processes. Gut bacteria and their metabolites may modulate specific aspects of brain function through immune, endocrine, metabolic, neural, and myelin-related mechanisms, while direct evidence for effects on human intelligence and consciousness remains limited. The HPA axis and the gut microbiota-oligodendrocyte axis form a bidirectional communication network that may shape brain structure and function. The HPA axis governs the body’s hormonal stress response, while the GMOA controls myelin production. Together, they influence cognition, learning, and mental health, though direct links to raw human intelligence or core consciousness remain theoretical. Since the hippocampus plays an important role in learning and stress regulation, it is important to study the role of gut microbiota on the limbic system and other brain centers. We need to adopt a holistic approach in examining the role gut bacteria play in brain functions. We need to reconsider how we study interspecies quorum sensing (QS), gut-bacteria-generated signaling molecules, bacterial communication with IECs, the ENS and CNS, and immune reactions. The relatively recent “4E cognition” concept is aimed at improving our understanding of how we think, learn, and remember. Intelligence, cognition, and consciousness are a holobiontic product in which gut bacteria are part of a larger network (Figure 5).

**Figure 5.**
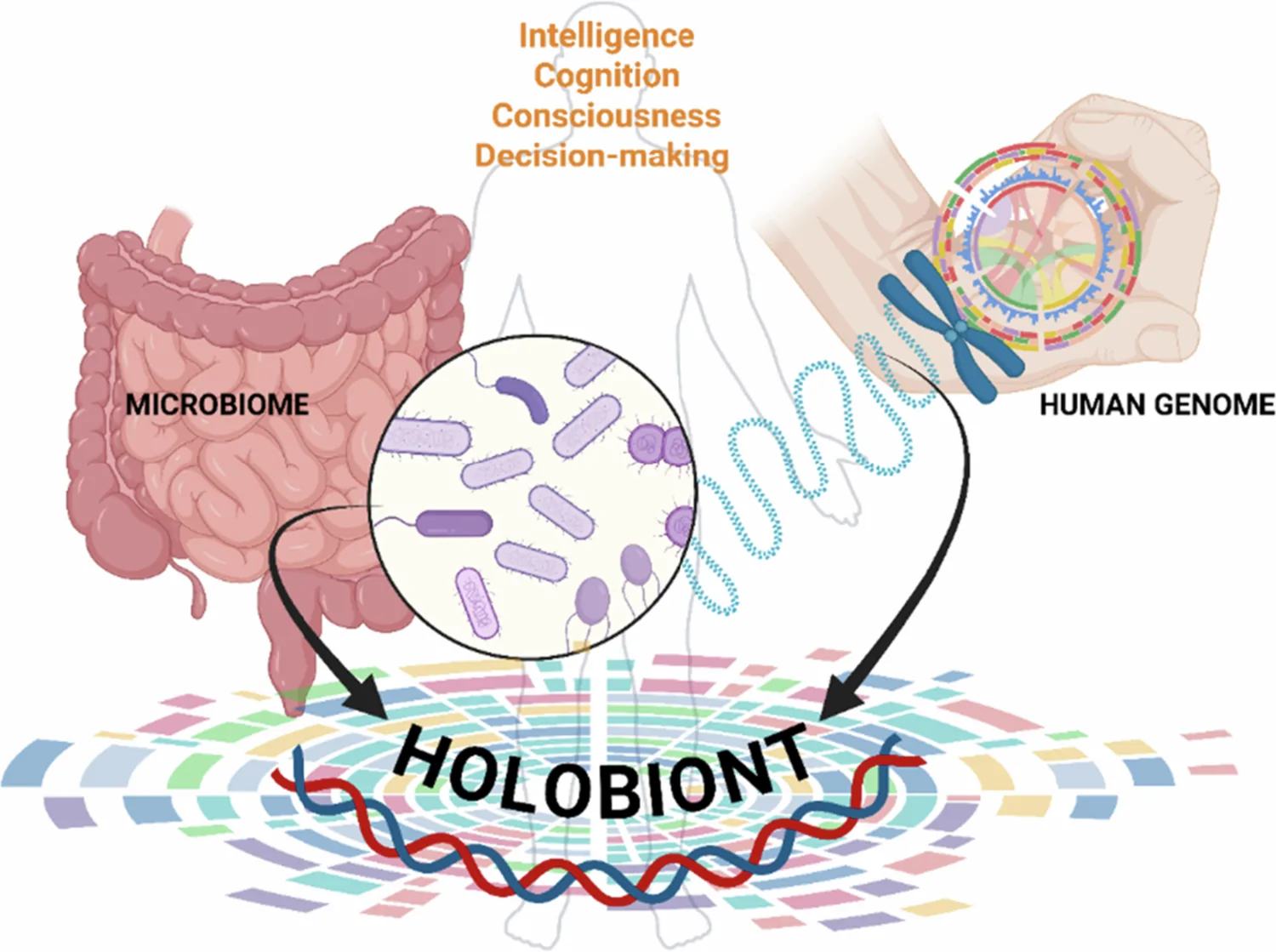
Intelligence, cognition, and consciousness are a holobiontic product in which gut bacteria are the main actors. The figure was prepared using Biorender.com (assessed 15 May 2026).

## Data Availability

Data used are from published papers available online.
